# Pervaporation Dehydration Mechanism and Performance of High-Aluminum ZSM-5 Zeolite Membranes for Organic Solvents

**DOI:** 10.3390/ijms25147723

**Published:** 2024-07-14

**Authors:** Qing Wang, Cheng Qian, Changxu Guo, Nong Xu, Qiao Liu, Bin Wang, Long Fan, Kunhong Hu

**Affiliations:** 1 School of Energy, Materials and Chemical Engineering, Hefei University, Hefei 230601, Chinachemhu@hfuu.edu.cn (K.H.); 2 State Key Laboratory of Materials-Oriented Chemical Engineering, College of Chemical Engineering, Nanjing Tech University, Nanjing 210009, China

**Keywords:** ZSM-5 zeolite membrane, organic solvent dehydration, pervaporation, secondary growth, adsorption–diffusion mechanism

## Abstract

Membrane-based pervaporation (PV) for organic solvent dehydration is of great significance in the chemical and petrochemical industries. In this work, high-aluminum ZSM-5 zeolite membranes were synthesized by a fluoride-assisted secondary growth on α-alumina tubular supports using mordenite framework inverted (MFI) nanoseeds (~110 nm) and a template-free synthesis solution with a low Si/Al ratio of 10. Characterization by XRD, EDX, and SEM revealed that the prepared membrane was a pure-phase ZSM-5 zeolite membrane with a Si/Al ratio of 3.8 and a thickness of 2.8 µm. Subsequently, two categories of PV performance parameters (i.e., flux versus separation factor and permeance versus selectivity) were used to systematically examine the effects of operating conditions on the PV dehydration performance of different organic solvents (methanol, ethanol, n-propanol, and isopropanol), and their PV mechanisms were explored. Employing permeance and selectivity effectively disentangles the influence of operating conditions on PV performance, thereby elucidating the inherent contribution of membranes to separation performance. The results show that the mass transfer during PV dehydration of organic solvents was mainly dominated by the adsorption–diffusion mechanism. Furthermore, the diffusion of highly polar water and methanol molecules within membrane pores had a strong mutual slowing-down effect, resulting in significantly lower permeance than other binary systems. However, the mass transfer process for water/low-polar organic solvent (ethanol, n-propanol, and isopropanol) mixtures was mainly controlled by competitive adsorption caused by affinity differences. In addition, the high-aluminum ZSM-5 zeolite membrane exhibited superior PV dehydration performance for water/isopropanol mixtures.

## 1. Introduction

The dehydration of organic solvents is crucial for obtaining highly purified organic solvents in the petrochemical, new energy (fuel), fine-chemical, and pharmaceutical industries [1,2]. Currently, mature large-scale purification techniques for organic solvents primarily rely on traditional distillation. However, this technique has disadvantages such as high energy consumption, large equipment footprint, and low purity (especially in azeotropic systems). Membrane-based pervaporation (PV) technology is acclaimed as one of the 21st-century high-precision separation technologies, praised for its low energy consumption, high efficiency, simple equipment, and easy control [3,4,5]. For instance, when producing an equal amount of product, membrane-based pervaporation consumes less than 10% of the energy required by distillation [6]. The properties of membrane materials are crucial in determining their separation performance. Researchers have developed various types of membrane materials, such as polymers, Metal–Organic Frameworks (MOFs), Covalent Organic Frameworks (COFs), SiC, and zeolites (molecular sieves) [7,8,9,10]. In particular, zeolite membranes have broad applications in membrane separation, membrane reactors, and electrode materials owing to their uniform pore size, excellent hydrothermal/chemical stability, as well as their impressive mechanical strength [11,12].

Over the past decades, various types of zeolite membranes, such as LTA (linde type A, pore size: 0.41 nm), CHA (chabazite, 0.38 nm), FAU (faujasite, 0.74 nm), MOR (mordenite, 0.65 × 0.70 nm), and MFI (mordenite framework inverted, 0.55 nm), have been developed and successfully utilized for the pervaporation separation of water/organic solvent mixtures [5,13]. Among various types of zeolite membranes, ZSM-5 membranes with MFI structure have attracted significant attention due to their distinctive pore structure, excellent hydrophilicity, acid stability, and catalytic activity. The micropore structure of ZSM-5 zeolite consists of sinusoidal channels (0.51 × 0.55 nm) along the *a*-axis interlinked with straight channels (0.53 × 0.56 nm) along the *b*-axis [14,15,16]. The Si/Al molar ratio of the ZSM-5 framework ranges from 3 to infinity [17,18]. Decreasing the Si/Al ratio enhances the hydrophilicity of the membrane, resulting in better performance in pervaporation dehydration [17,19,20].

The preparation of ZSM-5 zeolite membranes typically employs the organic template method, but this approach has some drawbacks. For example, during high-temperature calcination to remove organic templates, the membrane layer is susceptible to defects like cracks due to the differing thermal expansion coefficients between the support and the membrane layer [17,21]. On the other hand, preparing ZSM-5 zeolite membranes using the template-free method offers advantages by effectively avoiding the use of expensive organic templates and reducing the risk of defects caused by calcination. There have been many attempts to obtain defect-free ZSM-5 membranes via this synthesis route for the dehydration process of organic solvents. Noack et al. prepared ZSM-5 zeolite membranes on α-alumina supports through a secondary growth method using a gel with a Si/Al ratio of 50 without template agents [22]. These membranes exhibited a total flux of 0.13 kg/(m^2^ h) with a separation factor of ≤3 for the pervaporation separation of a 5/95 vol% water/methanol solution at 60 °C and a total flux of 0.14 kg/(m^2^ h), with a separation factor of 501 for a 5/95 vol% water/isopropanol solution at 80 °C. Subsequently, Li et al. successfully fabricated ZSM-5 zeolite membranes with a Si/Al ratio of 10.3 using a fluoride method without an organic template [17]. Li’s ZSM-5 membrane demonstrated excellent permeation performance for 10 wt% water/alcohol mixtures. For instance, it achieved a flux of 4.58 kg/(m^2^ h) for the PV dehydration of i-butanol with a separation factor exceeding 10,000 at 75 °C. Recently, Si et al. prepared a ZSM-5 zeolite membrane on an α-alumina support using a secondary hydrothermal synthesis method with a fluoride-containing gel that was template-free and had an extremely low Si/Al ratio (Si/Al = 9.09) [20]. Their membranes exhibited a flux of 0.451 kg/(m^2^ h) for a 10 wt% water/acetic acid mixture with a separation factor of 8991. The aforementioned research findings indicated that fluorides, acting as mineralizers, provided additional nucleation sites and promoted the growth of membrane layers. Additionally, preparing ZSM-5 zeolite membranes with a low Si/Al ratio (high aluminum) is an effective approach to achieving high permselectivity.

In previous work, we employed an organic template-assisted secondary growth to fabricate MFI zeolite membranes with an infinite Si/Al ratio. These membranes demonstrated superior separation performance for n-butane/i-butane mixtures and have been successfully applied in industrial settings [23]. However, preparing pure-phase ZSM-5 zeolite membranes with low Si/Al ratios without template agents is rather challenging. For instance, within a low Si/Al ratio range of 15 to 40, the fabricated ZSM-5 zeolite membranes often contain a significant number of amorphous substances or competing phase impurities, such as mordenite [24,25,26]. Additionally, small seeds with a high surface area for secondary growth could provide abundant nucleation sites, significantly promoting the growth of membrane layers [27,28]. Therefore, this study utilized MFI nanoseeds to prepare ZSM-5 membranes with low Si/Al ratios on α-Al_2_O_3_ tubular supports using a fluoride-assisted secondary growth method without organic templates. The synthesized ZSM-5 zeolite membranes were further applied in the PV separation of water/organic solvent mixtures. A systematic investigation was conducted on the effects of operating conditions on the pervaporation dehydration performance of methanol (MeOH), ethanol (EtOH), n-propanol (n-PrOH), and isopropanol (IPA). Additionally, the separation mechanisms of PV dehydration using high-aluminum ZSM-5 zeolite membranes were explored.

## 2. Results and Discussion

### 2.1. Characterization of MFI Nanoseeds and Seed Layers

**Figure 1:**
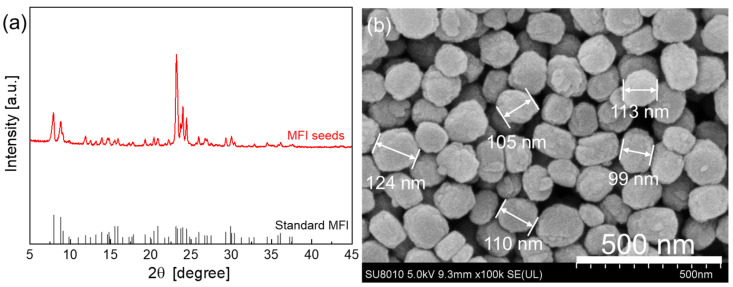
(**a**) XRD pattern and (**b**) SEM image of the as-prepared nanoseeds.

Figure 1 displays the XRD pattern and SEM image of the prepared MFI nanocrystal seeds. The XRD pattern exhibited a series of characteristic peaks, such as at 2θ = 7.95°, 8.84°, and 23.14°, which matched well with the standard MFI [14,18,20], indicating that the prepared zeolite was a pure-phase MFI nanoseed. The SEM image revealed that the morphology of the prepared MFI seeds was approximately “spherical” without twinning. The seeds exhibited a very uniform size of approximately 110 nm. Thus, pure-phase MFI nanoseeds were successfully synthesized and subsequently used to prepare the seed layer.

**Figure 2:**
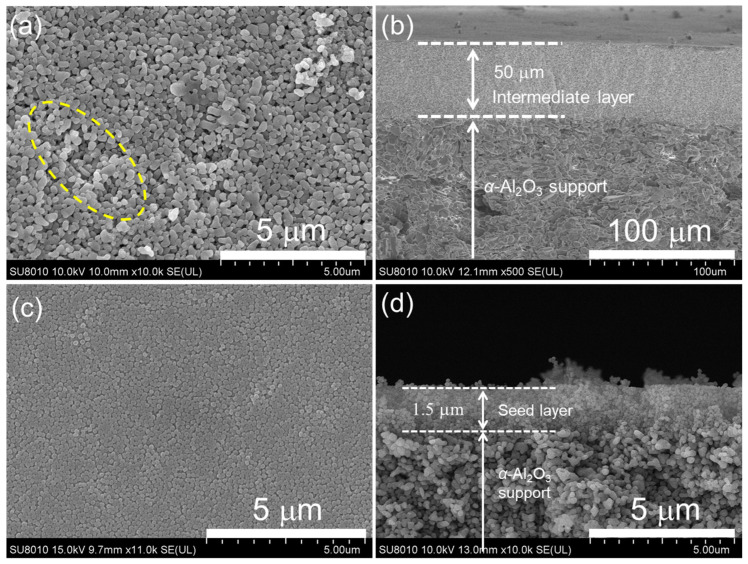
The typical surface and cross-sectional SEM images of (**a**,**b**) the pristine support and (**c**,**d**) the final seeded support. The micron-sized pores on the support surface are highlighted with yellow ellipses.

In secondary growth, seeds could decouple the nucleation and growth processes of zeolites by providing crystallization nuclei, significantly shortening the membrane preparation time [29]. Furthermore, a continuous and dense seed layer could reduce the negative influence of the surface roughness of the support on the membrane layer, thereby reducing the generation of defects [28]. However, the quality of the seed layer is particularly crucial for preparing continuous and defect-free ZSM-5 zeolite membranes. Figure 2a,b show the surface and cross-sectional SEM images of the asymmetric α-alumina tubular support used in this work. It was observed that the support surface was relatively rough, with micron-sized pores (highlighted by a yellow ellipse). Figure 2c,d show SEM images of the seed layer prepared on the support with a dip-coating method. The support surface was covered with a continuous, smooth, and dense seed layer with a thickness of 1.5 µm. The coated support will undergo hydrothermal conditions to grow into a membrane.

### 2.2. Preparation and Characterization of ZSM-5 Membranes

**Figure 3:**
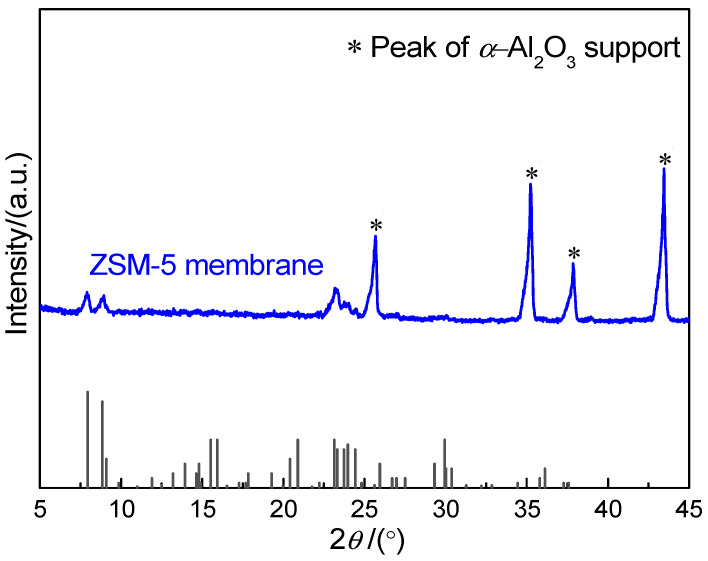
XRD pattern of the prepared ZSM-5 membrane.

Figure 3 shows the XRD pattern of the ZSM-5 membranes hydrothermally synthesized at 165 °C for 24 h using a template-free solution with a low Si/Al ratio of 10. The XRD result of the membrane indicated that, except for the diffraction peaks of the alumina support, all other diffraction peaks were consistent with the standard MFI. Moreover, the Si/Al ratio of the membrane was determined by EDX to be 3.8. The results suggest that pure-phase, aluminum-rich ZSM-5 membranes were successfully prepared.

**Figure 4:**
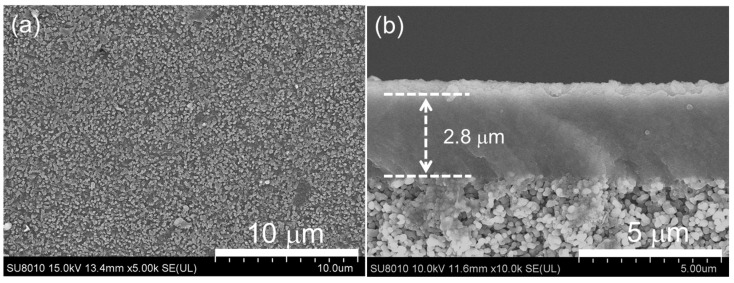
(**a**) Surface and (**b**) cross-sectional SEM images of the synthesized ZSM-5 membrane.

The microstructure of the membrane was observed using SEM, and the results are shown in Figure 4. Figure 4a shows needle-like crystals on the membrane surface, which is a typical morphology observed in high-alumina ZSM-5 membranes. Additionally, no gel-like amorphous substances were observed, indicating that the prepared membrane had good crystallinity. Figure 4b demonstrates that the crystals were intergrown, forming a continuous and dense membrane layer without significant defects, with a thickness of approximately 2.8 µm. Notably, under similar preparation conditions, MFI zeolite membranes derived from micron-sized seeds tend to possess defects such as voids, as reported in the literature [30,31]. In contrast, nano-sized seeds are more conducive to forming dense and intergrown membranes. This is plausibly attributed to the smaller gaps and higher packing density between the nanocrystals in the seed layer, which reduces defects in the membrane layer.

### 2.3. Pervaporation Dehydration Performance of ZSM-5 Membranes

This section systematically investigated the effects of operating conditions (feed temperature and concentration) on the pervaporation dehydration performance of MeOH, EtOH, n-PrOH, and IPA through aluminum-rich ZSM-5 membranes. The PV permeation mechanism was also discussed. It is well known that there are two systems of parameters used to evaluate PV performance: (1) flux vs. separation factor and (2) permeance vs. selectivity. The former is a widely used parameter system due to its simplicity and ease of calculation. However, with the continuous development of computer software (e.g., Aspen) and the availability of various physical data, the latter is also gradually gaining attention. The values of permeation flux and separation factor are influenced by both the intrinsic properties of membrane materials and the operating conditions (e.g., feed temperature and concentration) in pervaporation [32]. This is attributed to the fact that the changes in activity coefficients (γ*_i_*) and saturated vapor pressures (*p_i_*^o^) of the components under different PV conditions lead to variations in the driving force (i.e., the partial pressure difference in Equation (3): *p_f_*_,*i*_ − *p_p_*_,*i*_ = $\gamma_{i}$*x_i_p_i_*_,_^o^) for mass transfer. However, using permeance and selectivity effectively decouples the influence of operating conditions on PV performance, thereby elucidating and quantifying the contribution of the intrinsic properties of the membrane to separation performance [33]. Therefore, the pervaporation dehydration performance was systematically evaluated here using two parameter systems.

#### 2.3.1. Effect of Operating Temperature

**Figure 5:**
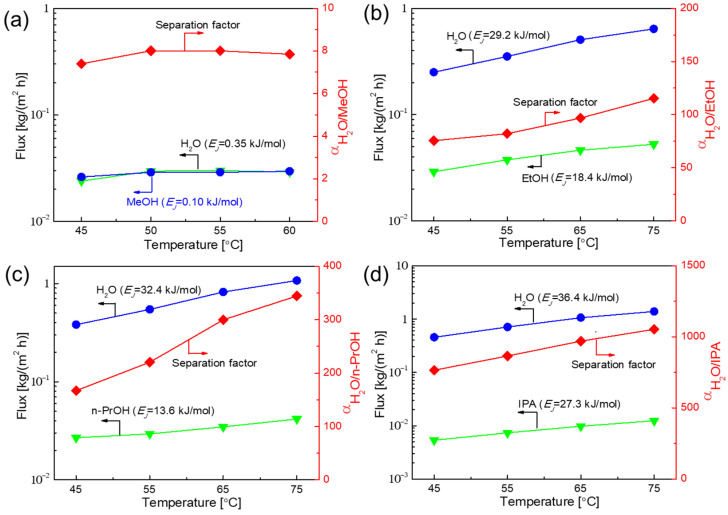
Effect of feed temperature on separation factors and flux through a ZSM-5 membrane for binary systems containing 10 wt% water: (**a**) H_2_O/MeOH, (**b**) H_2_O/EtOH, (**c**) H_2_O/n-PrOH, and (**d**) H_2_O/IPA.

The operating temperature is one of the critical parameters that significantly influences the performance of pervaporation dehydration. Figure 5 shows the effect of operating temperature on the flux and separation factor for binary mixtures of 10 wt% water and 90 wt% organic solvents (MeOH, EtOH, n-PrOH, and IPA). It could be observed that in the water/MeOH binary system, both the flux and separation factor tended to increase with rising temperature. This increase was particularly significant for binary mixtures of water and EtOH, n-PrOH, and IPA. The increase in flux with temperature was attributed to the enhanced driving forces for mass transfer resulting from the increased partial pressures of each component on the feed side and maintaining constant pressures on the permeate side [18,34]. The dependence of the separation factor on feed temperature could be elucidated by the apparent activation energy (*E_J_*_,*i*_) derived from the Arrhenius equation based on permeation flux, as shown below.

$$J_{i}=J_{0,i\;}exp\left(-\frac{E_{J,i}}{RT}\right)$$

In the given Equation, *J_i_*, *J_0_*_,*i*_, *R*, and *T* represent the permeation flux [kg/(m^2^ h)], pre-exponential factor [kg/(m^2^ h)], gas constant [8.314 kJ/(mol K)), and operating temperature [K], respectively. The apparent activation energy values calculated for each component are inserted in Figure 5. A positive activation energy value signifies that the flux increases with an increase in temperature and vice versa [4]. Examination of each binary system revealed that water exhibited higher apparent activation energy than organic solvents. This means that as the temperature increased, the water flux increased faster than that of organic solvents, leading to an increase in the separation factor of the water/organic solvents. Therefore, each binary system achieved the highest permeation flux and separation factor at high operating temperatures. Specifically, the separation factors for water/MeOH, water/EtOH, water/n-PrOH, and water/IPA were approximately 8 at 60 °C, 115 at 75 °C, 345 at 75 °C, and 1053 at 75 °C, respectively, with corresponding total fluxes of 0.06, 0.69, 1.12, and 1.40 kg/(m^2^ h). Notably, the total flux and separation factor values of the water/methanol system were more than an order of magnitude lower than those of the other systems. This suggests that the permeation behavior of water and methanol through the ZSM-5 membrane differed from that of the other systems, which is further discussed later in terms of permeance and selectivity.

**Figure 6:**
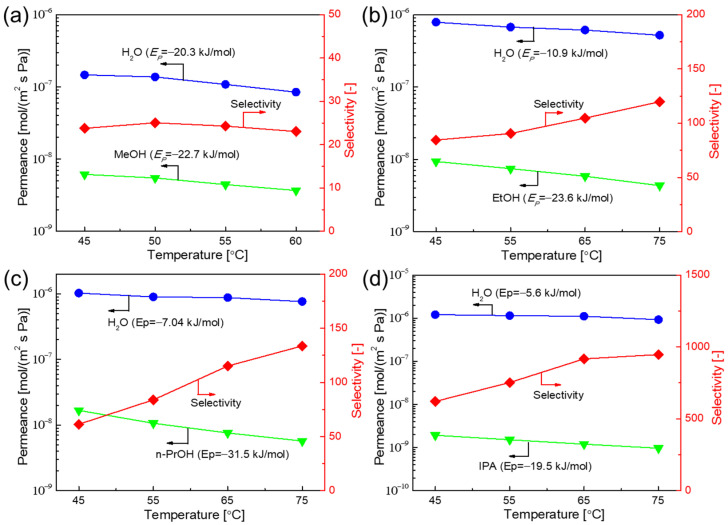
Effect of feed temperature on permeance and selectivity through a ZSM-5 membrane for binary systems containing 10 wt% water: (**a**) H_2_O/MeOH, (**b**) H_2_O/EtOH, (**c**) H_2_O/n-PrOH, and (**d**) H_2_O/IPA.

Figure 6 illustrates the influence of operating temperature on permeance and selectivity in the corresponding binary systems. The permeance of both water and organic solvents gradually decreased with temperature. However, except for the water/methanol system, where selectivity tended to remain constant, the selectivity of other water/organic solvent systems significantly increased. The temperature dependence of permeance is also investigated using the permeation activation energy (*E_P_*_,*i*_) derived from the Arrhenius equation [4,35], as follows.

$$P_{i}=P_{0,i\;}exp\left(-\frac{E_{P,i}}{RT}\right)$$

In the given Equation, *P_i_* and *P*_0,*i*_ represent the permeance [mol/(m^2^ s Pa)] and pre-exponential factor [mol/(m^2^ s Pa)], respectively. The calculated results of the permeation activation energy are presented in Figure 6. The permeation activation energies of water and the organic solvent were negative in each binary system, suggesting an adsorption-dominated permeation behavior based on the adsorption–diffusion mechanism [4,28,36]. In the water/EtOH, water/n-PrOH, and water/IPA systems, the absolute values of the permeation activation energies of the organic solvents were significantly higher than those of water, indicating that the permeance of low-polarity (affinity) organic solvents was more sensitive to operating temperature. That is, the adsorption of water and organic solvents by the membrane declined with temperature; however, the decline was more significant for organic solvents. Accordingly, the selectivity of these binary systems increased significantly with temperature. Similar permeation behaviors were also observed during the PV dehydration of organic solvents using hydrophilic SiC-based membranes [4] and FAU membranes [28].

For the water/MeOH binary system, the permeation activation energies of water (*E_P_* = −20.3 kJ/mol) and methanol (*E_P_* = −22.7 kJ/mol) were similar, indicating that their permeance decreased to a comparable extent with temperature. Therefore, the selectivity changed little or tended to remain constant with temperature. Furthermore, the water permeance in the water/MeOH binary system exhibited a notably lower value compared to the other binary systems. A possible reason is that methanol molecules, which have stronger hydrophilicity due to a higher polarity index of 0.77 (as shown in Table 1) compared to the other three organic solvents (EtOH, n-PrOH, and IPA), are more easily adsorbed onto the membrane surface. Additionally, the MeOH molecules (0.3803 nm) are smaller than the membrane pores (~0.55 nm), making it easier for them to enter the membrane pores. However, the diffusion rate of MeOH was inevitably slower than that of small-sized water (0.2955 nm). Moreover, the strong hydrogen bonding interactions between water and methanol led to a strong mutual deceleration effect on their diffusion within the membrane pores [37,38]. Consequently, the flux and permeance in the binary system of water/MeOH were significantly lower than in the other systems. This phenomenon was consistent with the result demonstrated by Chen et al. through molecular simulations [39]. In contrast, EtOH, n-PrOH, and IPA have relatively low polarity indices (low affinities), resulting in their adsorption on the membrane surface being inhibited by preferentially adsorbed water (i.e., competitive adsorption). Additionally, their larger molecular sizes are somewhat restricted from freely entering the membrane pores, thus leading to higher water permselectivity. The above results also indicate that high-aluminum ZSM-5 membranes exhibited good PV separation performance for the water/IPA system. Therefore, the influence of feed concentration on PV separation performance will be discussed using H_2_O/IPA mixtures as an example.

#### 2.3.2. Effect of Feed Concentration

**Figure 7:**
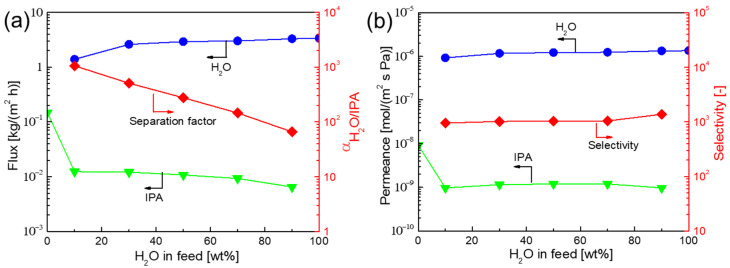
The influence of feed concentration in water/IPA mixtures on (**a**) flux and separation factor and (**b**) permeance and selectivity.

Figure 7 illustrates the influence of feed concentration on the PV performance of ZSM-5 membranes for the water/IPA binary system at 75 °C. As depicted in Figure 7a, the water flux increased with feed concentration, whereas the IPA flux and the separation factor showed the opposite trend. This phenomenon occurred due to the increase in the partial pressure of water with feed concentration, enhancing the driving force for the mass transfer of water, and the opposite was true for IPA. The reduction in the separation factor could be described using Equation (2): the water flux or the water concentration on the permeate side did not increase linearly with feed concentration [4,28]. Figure 7b illustrates the effect of feed concentration on the permeance and selectivity of water/IPA mixtures through the ZSM-5 membrane. In the binary mixture region, the water permeance showed a slight increase with feed concentration, whereas the IPA permeance either remained nearly constant or tended to decrease, leading to a gradual improvement in selectivity. Notably, the IPA permeance in the binary system was significantly lower than that in pure IPA solution. This was plausibly attributed to the high hydrophilicity of the ZSM-5 membrane, which exhibited a much stronger affinity for high-polar H_2_O molecules (polarity index = 1) compared to the low-polar IPA (polarity index = 0.38). As a result, H_2_O molecules adsorbed preferentially on the surface of membranes, thereby hindering the adsorption of IPA (i.e., competitive adsorption). The influence of feed concentration on separation performance further revealed that the pervaporation dehydration of organic solvents through ZSM-5 membranes was dominated by an adsorption–diffusion mechanism.

### 2.4. Comparing Pervaporation Performance and Reproducibility of ZSM-5 Membranes

**Figure 8:**
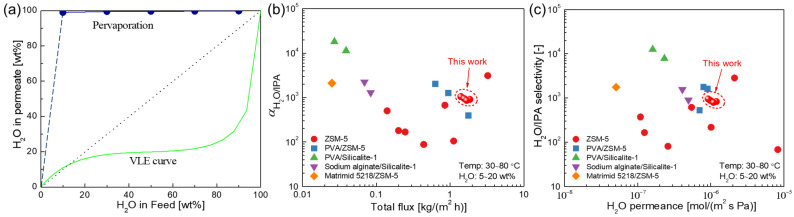
(**a**) Comparison of PV selectivity with the distillation selectivity derived from the vapor–liquid equilibrium (VLE) curve for water/IPA at 75 °C; comparison of the separation performance of water/IPA mixtures using PV membranes (ZSM-5 [21,22,43] and MFI-zeolite-containing mixed matrix membrane [44,45,46]) under similar test conditions: (**b**) flux versus separation factor and (**c**) permeance versus selectivity.

Due to the existence of azeotropes, efficiently separating water and IPA mixtures is currently a major challenge in industrial production. Figure 8a compares the PV selectivity with the distillation selectivity derived from the vapor–liquid equilibrium (VLE) of the water/IPA system at 75 °C to evaluate the PV separation capability of ZSM-5 membranes. The diagonal line in the figure denotes a selectivity of 1 (i.e., non-selectivity for azeotropic mixtures). As the deviation of the PV or VLE curve increases from this line, the selectivity towards water or IPA increases. Obviously, PV exhibited superior selectivity compared to distillation (VLE) and demonstrated efficient separation performance around the azeotropic point (12.7 wt% water/IPA). Moreover, the water content in the PV permeates exceeded 99 wt%, a separation efficiency that is difficult to achieve with traditional distillation.

The pervaporation dehydration performance of ZSM-5 membranes was compared with data in the literature [21,22,43,44,45,46] under comparable test conditions, as illustrated in Figure 8b,c. Compared to other ZSM-5 membranes and mixed matrix membranes containing MFI zeolite (silicalite-1 or ZSM-5) as reported in the literature, the ZSM-5 membrane prepared in this work exhibited superior PV performance for water/IPA in both systems of PV evaluation parameters: flux versus separation factor and permeance versus selectivity. For example, the as-prepared ZSM-5 membranes occupied a technologically appealing region, achieving a high total flux of ~1.40 kg/(m^2^ h) and a separation factor of 1053 at 75 °C. Likewise, the corresponding water permeance was ~1.0 × 10^−6^ mol/(m^2^ s Pa) with a selectivity of 947. This clearly demonstrates the great potential of high-aluminum ZSM-5 membranes for PV separation of water/IPA mixtures. It is worth noting that each data point in Figure 8b or Figure 8c represents the PV performance of ZSM-5 membranes prepared in different batches under identical conditions. Clearly, the data in this study exhibited good convergence, showing similar levels of flux (or permeance) and separation factor (or selectivity), thereby demonstrating the reproducibility of the membrane fabrication. These results reveal that the high-performance, reproducible, high-aluminum ZSM-5 membranes developed without organic templates have great potential in the PV dehydration of organic solvents.

## 3. Materials and Methods

### 3.1. Materials

**Scheme 1:**
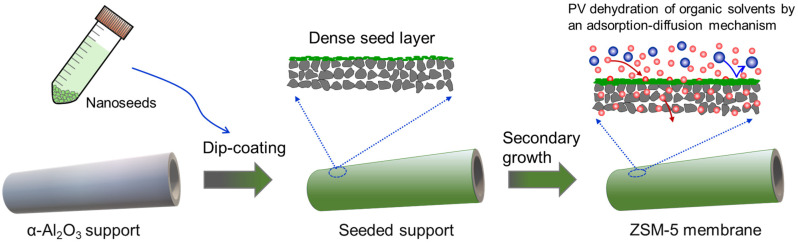
Preparation route and application of high-aluminum ZSM-5 membranes for PV dehydration of organic solvents.

Colloidal silica (HS-40, 40 wt%, Aldrich, Shanghai, China) and tetraethoxysilane (TEOS, 98 wt%, Greagent, Shanghai, China) were used as silicon sources. Aluminum sulfate hydrate (Al_2_(SO_4_)_3_·18H_2_O, 99 wt%, Greagent, Shanghai, China) was used as an aluminum source. Tetrapropylammonium hydroxide (TPAOH, 25 wt%, Damas-beta, Shanghai, China) was used as an organic template agent for seed synthesis. Sodium fluoride (NaF, 99 wt%, Greagent, Shanghai, China) and sodium hydroxide (NaOH, 98 wt%, Greagent, Shanghai, China) were also used. The porous α-Al_2_O_3_ tubular supports (average pore size 200 nm, outer diameter 12 mm, wall thickness 2 mm, length 60 mm) were purchased from Nanjing Tech University. The preparation route of ZSM-5 zeolite membranes is shown in Scheme 1.

### 3.2. Synthesis of MFI Nanoseeds and Seeded Supports

The MFI (silicalite-1) nanoseeds were prepared by modifying our previously reported procedure [14]. In brief, TPAOH and deionized water were first mixed, followed by the gradual dropwise addition of TEOS to the mixture, resulting in a synthesis solution with a molar composition of TEOS: 0.2TPAOH: 19.2H_2_O. The synthesis solution was transferred to an autoclave and then synthesized at 95 °C for 48 h. The resulting milky suspension was subjected to high-speed centrifugation, washing, and drying to obtain MFI crystals. It is worth noting that an ethanol solution was chosen to disperse the seeds in order to facilitate the complete dispersion of hydrophobic silicalite-1 seeds. A 0.25 wt% seed suspension was prepared. As previously reported [14,28,47], the nanoseeds were deposited on the outer surface of supports via a dip-coating process and subsequently dried at 80 °C for 0.5 h.

### 3.3. Preparation of ZSM-5 Membranes

ZSM-5 zeolite membranes were prepared on α-Al_2_O_3_ tubular supports using a fluoride-assisted secondary growth method without an organic template. The synthesis solution for preparing ZSM-5 zeolite membranes, with a molar composition of SiO_2_: 0.05Al_2_O_3_: 0.20Na_2_O: 0.99NaF: 50H_2_O, was obtained by mixing deionized water, NaOH, HS-40, Al_2_(SO_4_)_3_·18H_2_O, and NaF. The resulting mixture was stirred and aged for 2 h. Subsequently, the seeded support was vertically fixed in an autoclave, and the synthesis solution was slowly added. The hydrothermal synthesis was conducted for 24 h at a temperature of 165 °C. After the reaction, the prepared membranes were washed with water and then stored at room temperature for later use.

### 3.4. Characterization and Pervaporation Experiments

The microstructures and elemental compositions of the samples were observed and analyzed using a field emission scanning electron microscope (SEM, SU8010) combined with energy-dispersive X-ray (EDX) spectroscopy. The crystalline structures of the seeds and zeolite membranes were analyzed by X-ray diffraction (XRD, TD-3500) with Cu Kα radiation in a 2θ range from 5 to 45°.

Pervaporation measurements were conducted on mixtures of water and organic solvents (methanol (MeOH), ethanol (EtOH), n-propanol (n-PrOH), and isopropanol (IPA)) with the physical properties of these molecules listed in Table 1. PV experiments were conducted utilizing a self-built apparatus, as reported earlier (Figure S1) [18,28]. The membrane was vertically submerged in the feed tank, with the feed side maintained at ambient pressure and the permeate side kept at approximately 0 kPa via a vacuum pump. The samples were collected using a cold trap immersed in liquid nitrogen, and their compositions were analyzed using a gas chromatograph (GC-9860). The flux [J, kg/(m^2^ h)], separation factor (α*_i_*_/*j*_), permeance [*P_i_*, mol/(m^2^ s Pa)], and selectivity of membranes were calculated using the following Equations (1)–(4).

(1) $$J_{t}=\frac{w}{A\;t}$$

(2) $$\alpha_{i/j}=\frac{y_{i}}{y_{j}}/\frac{x_{i}}{x_{j}}$$

(3) $$P_{i}=\frac{J_{m,i}}{p_{f,i}-p_{p,i}}$$

(4) $$Selectivity=\frac{P_{i}}{P_{j}}$$

where *w* represents the mass of the collected sample during the collection time *t* across the membrane area *A*, and *x* and *y* are the mole fractions of component *i* (or *j*) in the feed and permeate solutions, respectively. A detailed description of the calculation of permeance using Equation (S3) is given in the Supplementary Materials.

## 4. Conclusions

In this work, high-aluminum ZSM-5 zeolite membranes were successfully synthesized at 165 °C for 24 h on α-Al_2_O_3_ tubular supports using MFI nanoseeds and a fluoride-assisted secondary growth method without organic templates. The micromorphology, Si/Al ratio, and crystalline phase structure of the membranes were characterized by SEM, EDX, and XRD. The results show that the pure-phase, high-aluminum ZSM-5 membrane had a thickness of 2.8 µm and a Si/Al ratio of 3.8. Subsequently, two categories of PV performance parameters (i.e., flux versus separation factor and permeance versus selectivity) were used to systematically examine the effects of operating conditions on the PV dehydration performance of methanol, ethanol, n-propanol, and isopropanol. Employing permeance and selectivity effectively disentangles the influence of operating conditions on PV performance, thereby elucidating the inherent contribution of membranes to separation performance. The results show that the mass transfer during PV dehydration of organic solvents was mainly dominated by the adsorption–diffusion mechanism. Furthermore, the diffusion of highly polar water and methanol molecules within the membrane pores had a strong mutual slowing-down effect, resulting in a significantly lower permeance than other binary systems. However, the mass transfer process for water/low-polarity organic solvent (ethanol, n-propanol, and isopropanol) mixtures was mainly controlled by competitive adsorption caused by affinity differences. In addition, the high-aluminum ZSM-5 membrane exhibited superior PV dehydration performance for water/isopropanol mixtures.

## Figures and Tables

**Table 1:** Molecular sizes [40] and physical properties of the molecules used in this study [41,42].

Solvents	Molar Mass [g/mol]	Molecular Size [nm]	Molar Volume [cm^3^/mol]	HSPs (MPa^1/2^)				Polarity Index [-]
				$\delta_{d}$	$\delta_{p}$	$\delta_{h}$	$\delta_{t}$	
water	18.02	0.2955	18.02	15.6	16.0	42.3	47.8	1.00
methanol	32.04	0.3803	40.49	15.1	12.3	22.3	29.6	0.77
ethanol	46.07	0.4299	58.4	15.8	8.8	19.4	26.5	0.55
n-propanol	60.10	0.4691	74.79	16.0	6.8	17.4	24.5	0.43
isopropanol	60.10	0.4699	76.55	15.8	6.1	16.4	23.5	0.38

Here, $\delta_{p}$, $\delta_{d}$, and $\delta_{h}$ are the polar, dispersive, and hydrogen bonding contributions, respectively, to the total Hansen solubility parameter $\delta_{t}$, ($\delta_{t}=\sqrt{\delta_{d}^{2}+\delta_{p}^{2}+\delta_{h}^{2}}$). The polarity index quantifies the polarity of organic solvents relative to water.

## Data Availability

Data will be made available on request.

## Supplementary Materials

The following supporting information can be downloaded at: https://www.mdpi.com/article/10.3390/ijms25147723/s1. Reference [48] are cited in the Supplementary Materials.
