# Supplementary material for: Pervaporation Dehydration Mechanism and Performance of High-Aluminum ZSM-5 Zeolite Membranes for Organic Solvents
Source: Int J Mol Sci. 2024 Jul 14;25(14):7723. doi: 10.3390/ijms25147723 (PMC11277172; doi:10.3390/ijms25147723)
Supplement: Supplementary file 1 [file ijms-25-07723-s001.zip › ijms-3091056-supplementary.pdf]

Supplementary Materials

Pervaporation Dehydration Mechanism and Performance of High-Aluminum ZSM-5 Zeolite Membranes for Organic Solvents

Qing Wang [1,2,*,†], Cheng Qian [1,†], Changxu Guo [1], Nong Xu [1,2,*], Qiao Liu [1], Bin Wang [2], Long Fan [1] and Kunhong Hu [1]

[1] School of Energy, Materials and Chemical Engineering, Hefei University, Hefei 230601, China
[2] State Key Laboratory of Materials-Oriented Chemical Engineering, College of Chemical Engineering, Nanjing Tech University, Nanjing 210009, China
[*] Correspondence: qingwang@hfuu.edu.cn (Q.W.); xunong@hfuu.edu.cn (N.X.).
[†] These authors contributed equally to this work.

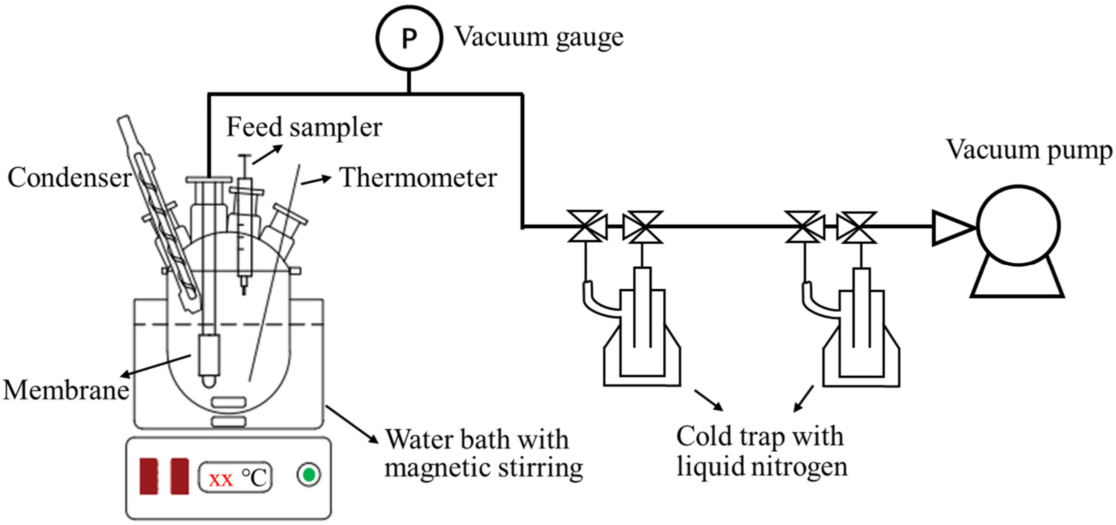

Figure S1. Schematic diagram of pervaporation apparatus.

Permeance calculation of pervaporation

The driving force of pervaporation was calculated by the difference in the partial pressures of the feed and permeate streams, and the permeance of the *i* component, P_i, was determined using Equation (S1).

$$P_i = J_{m,i}/(p_{f,i} - p_{p,i}) \tag{S1}$$

where J_i, $p_{f,i}$, and $p_{p,i}$ are the molar flux of the *i* component, the feed stream partial pressure, and the permeate stream partial pressure, respectively. $P_{f,i}$, was calculated as $p_{f,i} = \gamma_i x_i p_{i,o}$ with vapor pressure, $p_{i,o}$, by Antoine equation (Equation (S2)), mole fraction, x_i, and activity coefficient, γ_i, by Wilson equation (Equation (S3)), while a permeate pressure, $p_{p,i}$, was assumed to be zero. The Antoine coefficients and Wilson parameters used for this study are listed in Table S1.

$$\log p_{i,o}\ [\text{mmHg}] = A - B/(t\ [°\text{C}] + C) \tag{S2}$$

where $p_{i,0}$ and t are partial pressure of the *i* component and temperature, respectively.

$$\ln\gamma_1 = -\ln(x_1 + \Lambda_{12}x_2) + x_2\left[\frac{\Lambda_{12}}{x_1 + \Lambda_{12}x_2} - \frac{\Lambda_{12}}{\Lambda_{21}x_1 + x_2}\right]$$

$$\ln\gamma_2 = -\ln(x_2 + \Lambda_{21}x_1) - x_1\left[\frac{\Lambda_{12}}{x_1 + \Lambda_{12}x_2} - \frac{\Lambda_{12}}{\Lambda_{21}x_1 + x_2}\right] \tag{S3}$$

where Λ_{12} and Λ_{21} are Wilson parameters.

Table S1. Antoine coefficients and Wilson parameters used in this study, where component 2 is water [1].

Component 1	Antoine coefficients (Component 1)			Antoine coefficients (Component 2)			Wilson parameters	
	A	B	C	A	B	C	Λ_{12}	Λ_{21}
methanol	7.87863	1473.110	230.000	8.10765	1750.286	235.000	0.48125	1.01184
ethanol	8.04494	1554.300	222.605	8.10765	1750.286	235.000	0.19291	0.65806
n-propanol	7.99733	1569.700	209.500	7.96681	1668.210	228.000	0.04793	0.61233
isopropanol	6.6604	813.055	132.930	7.96681	1668.210	228.000	0.03084	0.78363

Reference

1. Hirata, M.; Ohe, S.; Nagahama, K. *Vapor-liquid equilibrium data calculated using electronic computers*; Kodansha Co: Tokyo, Japan, 1975.

Disclaimer/Publisher's Note: The statements, opinions and data contained in all publications are solely those of the individual author(s) and contributor(s) and not of MDPI and/or the editor(s). MDPI and/or the editor(s) disclaim responsibility for any injury to people or property resulting from any ideas, methods, instructions or products referred to in the content.
